# Sonic Hedgehog Agonist Protects Against Complex Neonatal Cerebellar Injury

**DOI:** 10.1007/s12311-017-0895-0

**Published:** 2017-11-13

**Authors:** Vien Nguyen, Khalida Sabeur, Emin Maltepe, Kurosh Ameri, Omer Bayraktar, David H. Rowitch

**Affiliations:** 1 Department of Pediatrics, Eli and Edythe Broad Institute for Stem Cell Research and Regenerative Medicine, University of California, San Francisco, 513 Parnassus Avenue, San Francisco, CA 94143 USA; 2 Biomedical Sciences Graduate Program, University of California, San Francisco, 513 Parnassus Avenue, San Francisco, CA 94143 USA; 3 Division of Neonatology, University of California, San Francisco, 513 Parnassus Avenue, San Francisco, CA 94143 USA; 4 Department of Cardiology, University of California, San Francisco, 513 Parnassus Avenue, San Francisco, CA 94143 USA; 5 Department of Paediatrics, Wellcome Trust-MRC Stem Cell Institute, Cambridge University, Cambridge, UK

**Keywords:** Hypoxia, Glucocorticoid, Shh signaling, HIF signaling, Cerebral palsy, Brain development

## Abstract

The cerebellum undergoes rapid growth during the third trimester and is vulnerable to injury and deficient growth in infants born prematurely. Factors associated with preterm cerebellar hypoplasia include chronic lung disease and postnatal glucocorticoid administration. We modeled chronic hypoxemia and glucocorticoid administration in neonatal mice to study whole cerebellar and cell type-specific effects of dual exposure. Chronic neonatal hypoxia resulted in permanent cerebellar hypoplasia. This was compounded by administration of prednisolone as shown by greater volume loss and Purkinje cell death. In the setting of hypoxia and prednisolone, administration of a small molecule Smoothened-Hedgehog agonist (SAG) preserved cerebellar volume and protected against Purkinje cell death. Such protective effects were observed even when SAG was given as a one-time dose after dual insult. To model complex injury and determine cell type-specific roles for the hypoxia inducible factor (HIF) pathway, we performed conditional knockout of *von Hippel Lindau* (VHL) to hyperactivate HIF1α in cerebellar granule neuron precursors (CGNP) or Purkinje cells. Surprisingly, HIF activation in either cell type resulted in no cerebellar deficit. However, in mice administered prednisolone, HIF overactivation in CGNPs resulted in significant cerebellar hypoplasia, whereas HIF overactivation in Purkinje cells caused cell death. Together, these findings indicate that HIF primes both cell types for injury via glucocorticoids, and that hypoxia/HIF + postnatal glucocorticoid administration act on distinct cellular pathways to cause cerebellar injury. They further suggest that SAG is neuroprotective in the setting of complex neonatal cerebellar injury.

**Electronic supplementary material:**

The online version of this article (10.1007/s12311-017-0895-0) contains supplementary material, which is available to authorized users.

## Introduction

Approximately 60,000 preterm infants weighing less than 1500 g are born each year in the USA. Although survival rates are improving, long-term complications in such very low birth weight (VLBW) infants are commonly observed that involve the respiratory, cardiovascular, intestinal, and central nervous systems [1]. Cerebral palsy and other neurological sequelae in VLBW infants are associated with damage to cerebral white matter tracts [2] and cerebellum [3]. Cerebellar abnormalities are found in 20% of VLBW infants and are characterized as hemorrhagic or hypoplastic [4]. Clinical features associated with cerebellar abnormalities include postnatal glucocorticoid exposure, intraventricular hemorrhage, and chronic lung disease [5]. In particular, chronic lung disease can lead to intermittent hypoxemia and is associated with cerebellar hypoplasia in MRI studies [6, 7].

Although the cerebellum acts principally in the regulation of neural circuits for motor control and coordination [8], it also has roles in control of higher order cognitive functions [9–11]. Regions of the brain, undergoing extensive neurogenesis, are particularly vulnerable to hypoxic and/or ischemic insults during the third trimester and early neonatal periods [6, 7]. In humans, the cerebellum undergoes rapid growth during the third trimester through the first year of life [3, 6], whereas in rodents this phase of development is primarily post-natal.

The major driver of cerebellar growth is proliferation of cerebellar granule neuron precursors (CGNPs) [12], which depends on Sonic hedgehog (Shh) signaling [12–18]. Shh is a secreted protein that inhibits the transmembrane repressor Patched, which in turn, de-represses activity of Smoothened (Smo), a G-protein coupled receptor. Smo activation in the cilia of CGNP upregulates target genes *Gli1* and *N*-*myc* that drive cell cycle progression [15–17]. Thus, mutations affecting Shh production in Purkinje cells or Smo function on CGNP result in cerebellar hypoplasia [19].

Postnatal glucocorticoids are administered to preterm infants for indications of severe chronic lung disease and hypotension [3, 20, 21]. In the preterm lung, glucocorticoids promote production of pulmonary surfactant protein B and regulate the inflammatory response by interacting with transcription factors, such as nuclear factor kappaΒ (NF-κΒ) and activated protein 1 [22–24]. Although glucocorticoids help promote lung surfactant production and lung epithelial differentiation [22, 25], and physiological concentrations of these hormones are essential for normal brain development [26], high level exposure to potent glucocorticoids in the postnatal period causes brain injuries, including impaired cognition, cerebral palsy, and cerebellar hypoplasia [3, 6, 26–31].

11β-hydroxysteroid dehydrogenase type 2 (11βHSD2), a NAD-dependent high affinity enzyme involved in the local metabolic inactivation of endogenous glucocorticoids into inert 11-keto derivatives, acts in opposition to 11βHSD type 1, which converts its substrate into active corticosterone. Dexamethasone and betamethasone can cross the placenta to the fetus because they have a low affinity for cortisol binding globulin and are not inactivated by 11βHSD2, which is expressed at high levels in the placenta. In contrast, corticosterone and prednisolone are susceptible to inactivation by 11βHSD2 activity. 11βHSD2 is expressed in the developing CNS, including cerebellar granule neuron precursors (CGNPs) [32] where its function is necessary for normal cerebellar development [33]. Indeed, Shh signaling is protective against prednisolone-induced cerebellar injury through upregulation of 11βHSD2 specifically in CGNPs.

Chronic lung disease, airway instability, and apnea of prematurity can lead to an intermittent hypoxemic environment in the brain, which has been shown to affect cortical development, oligodendrocytes [34], and interneurons [35–37]. Certain cellular responses to hypoxia are mediated by hypoxia-inducible factors (HIFs) [38, 39], which are transcription factors with an unstable subunit (HIF1α or HIF2α) that is degraded in the presence of oxygen, and a constitutively expressed subunit (HIF1β or HIF2β) [40, 41]. HIFs coordinate the response to low oxygen by stimulating genes involved in metabolism and angiogenesis. In normoxia, HIFα becomes modified by prolyl-hydroxylase (PHD) and is recognized by the E3 ubiquitin ligase von Hippel Lindau factor (VHL), which then targets HIFα to the proteasome for degradation [42, 43]. Conversely in hypoxia, PHD is inactive, allowing HIFα to become stabilized, which then binds to HIFβ in the nucleus to activate target genes such as VEGF, BNIP3, glycolytic enzymes, and LEF-1/Tcf-1 [44, 45]. Previous studies show that glucocorticoid administration in neonatal mice causes cerebellar hypoplasia by downregulating Shh signaling and CGNP proliferation [29, 30] as well as cell death [46]; moreover, these effects are rescued by systemic administration of a small-molecule Smoothened agonist (SAG) [29]. The effect of hypoxia on cerebellar growth and its relationship to Shh signaling, however, is unclear. Moreover, cell type-specific roles of HIF in the developing cerebellum have so far not yet been defined.

Because exposure to glucocorticoid and hypoxia are clinically associated with cerebellar abnormalities [29, 30], we modeled such compound injury in neonatal mice using a chronic hypoxia model (10% FiO_2_) combined with administration of the synthetic glucocorticoid prednisolone. Although chronic hypoxia would model a relatively extreme form of clinical injury, it represents a robust and reproducible injury model that has been previously used and described extensively in the literature [34, 47, 48]. Intermittent hypoxia, while clinically more relevant, may confound results as it can precondition animals to neuroprotection from further hypoxic damage. We chose to use prednisolone rather than dexamethasone, which is more commonly given to infants with chronic lung disease, as dexamethasone is not metabolized by 11βHSD2 and therefore not affected by SAG [30]. As both prednisolone and hydrocortisone as sensitive to 11βHSD2, SAG therapy is therefore effective against these compounds.

Here we address the novel aspect of cross-talk between hypoxia/HIF signaling and glucocorticoid pathways in the developing cerebellum. We observed that chronic hypoxia resulted in cerebellar hypoplasia but that Purkinje cell populations were well preserved. When combined, however, hypoxia and glucocorticoids caused Purkinje cell death and enhanced cerebellar volume deficits. Both hypoplasia and Purkinje cell death were rescued in part by administration of SAG, even when given days after administration of the dual insults. To determine cell type-specific roles of the HIF pathway, we performed conditional knockout of VHL to induce and hyperactivate HIF1α in CGNPs or Purkinje cells. In the presence of active HIF in CGNP, prednisolone administration resulted in cerebellar hypoplasia. In contrast, prednisolone with active HIF in Purkinje cells resulted in cell death. Together, these findings indicate that hypoxia/HIF with postnatal glucocorticoid administration act on distinct cellular pathways to cause cerebellar injury. In particular, these results demonstrate for the first time the ill effects of HIF and glucocorticoid signaling in Purkinje cells. They further suggest that SAG is neuroprotective in the setting of complex neonatal cerebellar injury.

## Materials and Methods

### Animals

All animal procedures were reviewed and approved by the Institutional Animal Care and Use Committee and Laboratory Animal Resource Center at University of California, San Francisco (UCSF). Mouse colonies were maintained in accordance with NIH and UCSF guidelines. *C57Bl6/J* mouse lines were obtained from the Jackson Laboratories. *Gli*-*luciferase* [49], *VHL floxed* [50], *Atoh1*-*cre* (*Math1*-*cre*) [51], and *L7*-*cre* [19] mice have been previously described.

### Preparation of SAG

Synthesis of SAG has been previously described [52]. SAG was dissolved in dimethyl sulfoxide (DMSO) to 5 mM and further diluted with normal saline or culture medium. These experiments used SAG as a free-base form. Vehicle controls comprised saline containing an equivalent concentration of SAG.

### Chronic Hypoxic Rearing and Systemic Administration of Prednisolone and SAG

Chronic hypoxic rearing was performed as previously described [35, 48, 53, 54]. Briefly, litters of the *C57Bl6*/*J* strain were culled to a size of, at most, ten pups and co-fostered with CD1 or Swiss Webster strain dams then reared at 10% O_2_ in a hypoxic chamber (Biospherix, Inc., Laconia, NY) from postnatal day 3 (P3) to P11. Tissue from P4, P7, P11, P22, or P40 was then harvested acutely for analysis. On P3, pups received daily intraperitoneal injections of prednisolone (0.67 g/kg body weight, Sigma-Aldrich), SAG (20 g/kg body weight), prednisolone in combination with SAG, or vehicle (DMSO), for 8 days, or the duration of the hypoxic experiment. For acute treatment, a one-time dose of SAG was given at P11.

### Tissue Processing and Immunohistochemistry

Following intracardial perfusion of 4% paraformaldehyde, tissue was post-fixed for 1–2 h at 4 °C, cryoprotected in 30% sucrose, and embedded in OCT. Frozen sections were cut on a cryostat (20 μm) and stored at −80 °C. For staining, sections were thawed and then rehydrated in PBS. If necessary, tissues underwent antigen retrieval with citrate buffer (pH 6.0) at 95 °C for 10 min. Sections were blocked in 10% goat or donkey serum in 0.1% TritonX-100-containing PBS (blocking solution). Primary antibodies were diluted in blocking solution, and tissues incubated at 4 °C overnight or 2-h room temperature. For primary antibodies, we used PH3 (mouse monoclonal, Cell Signaling), Calbindin (mouse monoclonal or rabbit polyclonal, Swant), Cleaved Caspase 3 (rabbit polyclonal, Cell Signaling), NeuN (mouse monoclonal, Millipore), Iba1 (rabbit polyclonal, Wako), HIF1a (rabbit polyclonal, Cayman Chemicals), BNIP3 (rabbit polyclonal, Cell Signaling), and Cre (rabbit polyclonal, Millipore). Following primary incubation, tissues were washed with 0.1% Tween20-containing PBS, then incubated with proper Alexa Fluor secondary antibodies (Invitrogen) in blocking solution for 1 h at room temperature. Sections were mounted with fluoromount containing DAPI (Southern Biotech).

### CGNP Primary Cell Culture, Transfection, and Luciferase Assay

Primary cultures were performed as previously described [30]. Briefly, either *C57Bl6*/*J* or *Gli*-*luciferase* pups were euthanized at P4 or P5, and their cerebella were dissected and dissociated. Cultures were maintained in serum-free medium containing only vehicle or ShhN for 24 h prior to treatments. Cultures were then transfected with a HIF1α overexpressing plasmid or an empty vector for 12 h using the Piggyback transposon system to allow high efficiency expression as previously described [55]. Cells were collected 12 h afterward for protein extract or luciferase activity using the Dual-Luciferase Reporter Assay System (Promega). For analysis of Shh targets under hypoxic conditions, cultures were incubated in a 1% O_2_ incubator for 24 h, or treated with dimethyloxalylglycine (DMOG) (Sigma), a PHD4 inhibitor that upregulates HIF.

### Western Blot

Preparation of protein extracts, immunoblots, and fluorescent detection was done as previously described using the Li-Cor Odyssey system (Li-Cor, Lincoln, NE) [34]. Antibodies used include HIF1α (rabbit polyclonal, Cayman Chemicals), Cyclin D1 (rabbit polyclonal, ThermoScientific), Patched 1 (goat polyclonal, Santa Cruz), Gli1 (rabbit polyclonal, Santa Cruz), Gli3 (goat polyclonal, R and D Systems), N Myc (mouse monoclonal, Millipore), and β-Actin (mouse ascites, Sigma).

### Single-Molecule Fluorescent In Situ Hybridization Against *Gli1* and *Atoh1*

To assess *Gli1* mRNA expression in the cerebellum, we used the RNAscope LS Multiplex Assay (Advanced Cell Diagnostics). P11 mouse brain sections were baked at 65 °C for 45 min, post-fixed in 4% paraformaldehyde for 15 min, and dehydrated prior to the assay. Multiplexed single-molecule fluorescent in situ hybridization (smFISH) against *Gli1* and *Atoh1* mRNA was performed on the BOND RX automated stainer (Leica) using the RNAscope reagents. The *Gli1* probe consists of 20 double-Z probes targeting nucleotides 25–1025 on the mouse transcript NM_010296.2 and was developed with the fluorophore Opal 520 (Perkin Elmer). The *Atoh1* probe consists of 20 double-Z probes targeting nucleotides 847–2088 on the mouse transcript NM_007500.4 and was developed in Opal 570.

### Microscopy and Quantification

Surface area of the entire cerebellum was calculated as previously described [30]. Briefly, the cerebellum from immunostained sections with DAPI was outlined, and surface area was measured in Adobe Photoshop CS6 using the Record Measurements function, with measurement scale set to match the objective lens from the microscope (e.g., for the Zeiss × 10 objective, 1 pixel = 0.65 μm). For area measurement and cell counting of immunohistochemistry (IHC) from sagittal brain sections of P4, P7, P11, and P22 wild-type and transgenic animals, tiled images comprising the entire cerebellar vermis were taken with a Zeiss AxioImager.Z2 microscope equipped with a motorized stage using either the × 10 or × 20 objective. The mean area per section for each animal was determined in mm^2^ from measurements of five parasagittal sections from the midline region of the cerebellum, and is used as a proxy for volume. Quantification of cerebellar cross-sectional area resulted from an average of *n =* 3 animals in normoxic and hypoxic conditions for P4 and P22; *n =* 3 in normoxic, and *n =* 4 in hypoxic conditions at P40. For the hypoxia + prednisolone experiments, quantification of the cerebellar vermis is resulted from *n =* 3 animals for the Nx and Hx conditions, and *n =* 5 animals for the Hx + prednisolone and Hx + prednisolone + SAG conditions. For experiments using transgenic animals, quantification of the cerebellar IGL resulted from *n* = 3 animals for the *Math1Cre*;*Vhl*(*fl*/+) and *Math1Cre*;*Vhl*(*fl*/*fl*) conditions, *n* = 7 for *Math1Cre*;*Vhl*(*fl*/+) + prednisolone condition, and *n* = 6 for the *Math1Cre*;*Vhl*(*fl*/*fl*) + prednisolone condition; *n* = 3 for *L7Cre*;*Vhl*(*fl*/+), *L7Cre*;*Vhl*(*fl*/*fl*), and *L7Cre*;*Vhl*(*fl*/+) + prednisolone conditions, and *n* = 4 for *L7Cre*;*Vhl*(*fl*/*fl*) + prednisolone condition. The area of the cerebellum was measured using either Adobe Photoshop CS6 or Stereo Investigator (MBF Biosciences).

The numbers of immunopositive or double-immunopositive cells were quantified by a blinded investigator using the Count function on Adobe Photoshop CS6, or the Spot Detection function on Imaris (Bitplane). The average cell count for each animal was determined from measurements of five to seven whole parasagittal sections from the midline region of the cerebellum. Quantification of Calbindin-positive cells resulted from *n =* 3 animals in normoxic and hypoxic conditions, *n =* 6 in the hypoxia + prednisolone condition, and *n =* 5 in the hypoxia + prednisolone + SAG condition.

### Statistical Analyses

For all quantified data, mean + SEM values are presented. Statistical analysis was performed using unpaired, two-tailed Student’s *t* tests, and with an ANOVA (single factor). For a significant difference (*p* < 0.05), a Tukey’s post-hoc test was performed (GraphPad Prism).

## Results

### Chronic Hypoxia Causes Cerebellar Hypoplasia and Decreased CGNP Shh Signaling in Neonatal Mice

**Fig. 1:**
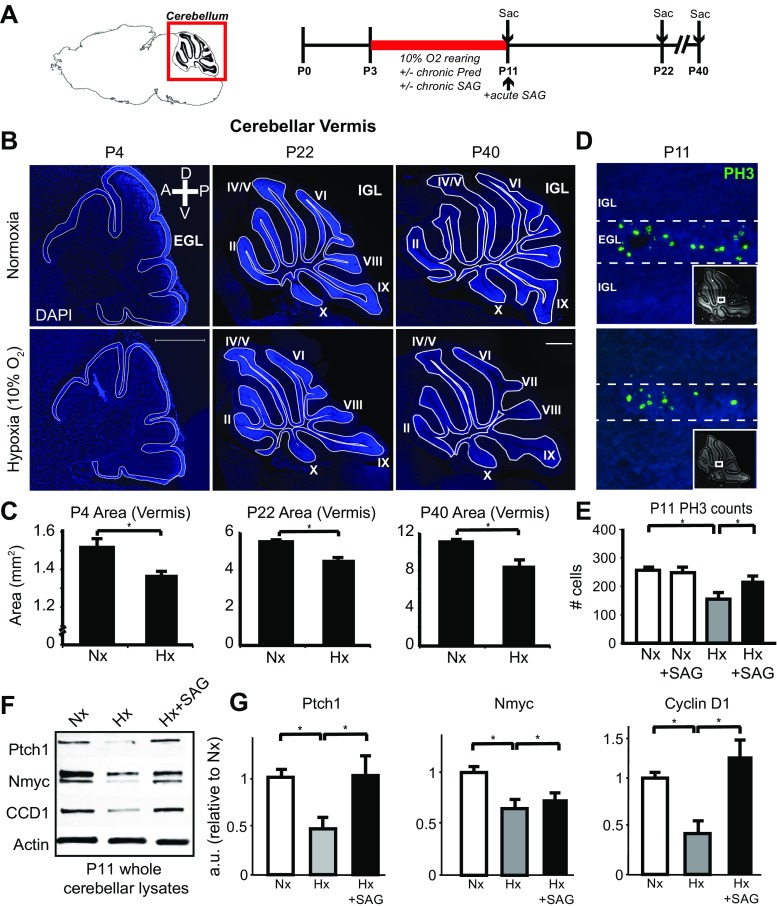
Chronic hypoxic rearing in postnatal mice results in permanent cerebellar hypoplasia through decreased CGNP proliferation and Shh signaling. **a** Schematic of anatomical region of hindbrain and cerebellum at vermis level, and experimental timeline for chronic hypoxic rearing, and for administration of prednisolone, SAG, or combination according to “chronic” (P3–P11) or “acute” (P11 only) schedule. **b** Representative images showing sagittal section of cerebellar vermis at different timepoints stained with DAPI. *A* anterior, *P* posterior, *D* dorsal, *V* ventral. Scale bar 500 μm. Outline of external granule cell layer (EGL) at P4 for localization of CGNP, and internal granule cell layer (IGL) at P22 and P40 for localization of CGNs. **c** Quantification of cerebellar cross-sectional area in normoxic (Nx) versus hypoxic (Hx) reared animals at P4, P22, and P40. At P4, area in Nx = 1.52 ± 0.0465 mm^2^ (*n =* 3), Hx = 1.37 ± 0.0181 mm^2^ (*n =* 3), *P* = 0.0432; P22, Nx = 5.49 ± 0.104 mm^2^ (*n =* 3), Hx = 4.56 ± 0.119 mm^2^ (*n =* 3), *P* = 0.00449; P40, Nx = 11.05 ± 0.1015 mm^2^ (*n =* 3), Hx = 8.527 ± 0.6121 mm^2^ (*n =* 4), *P* = 0.018. **d** Representative images showing proliferation in the EGL analyzed at P11 by PH3 staining; arrows denote PH3+ cells in the EGL; insert, representative area seen in whole cerebellum. **e** Quantification of PH3-positive cells in the EGL. Nx = 258 ± 11.9 cells (*n =* 3), Nx + SAG = 250 ± 9.63 cells (*n =* 3), Hx = 151 ± 25.1 cells (*n =* 4), Hx + SAG = 219.5 ± 18.2 cells (*n =* 3), *p* = 0.002. **f** Protein analysis of Shh target genes by Western blot; representative blots showing Patched1 (Ptch1), Nmyc, and Cyclin D1 (CCD1) levels under normoxic (Nx), hypoxic (Hx), and hypoxic + SAG (Hx + SAG) conditions. **g** Quantification of Shh proteins. Ptch1, Nx = 1 ± 0.0765, Hx = 0.470 ± 0.118, Hx + SAG = 1.0176 ± 0.282. Nmyc, Nx = 1 ± 0.0611, Hx = 0.649 ± 0.090, Hx + SAG = 0.740 ± 0.114. CCD1, Nx = 1 ± 0.0647, Hx = 0.424 ± 0.128, Hx + SAG = 1.226 ± 0.326. For quantification, mean + SEM; *n* ≥ 3 experiments/condition; *p < 0.05 Student’s *t* test

To study the effect of low oxygen tension on development of the brain, wild-type C57B6/J females with new litters were placed in a hypoxic (10% FiO_2_) cage for 7 days from postnatal days (P) 3–11 (Fig. 1a), a model that has previously been used to study reproducible phenotypes, including delayed myelination [34, 47]. After 24 h of hypoxia, where pups were reared in the chamber from P3 to P4, we observed reduced cerebellar size that was significant (Fig. 1b, c; P4 Nx vs. Hx, *p* = 0.0432; EGL outlined for clarity) despite lower body weight of hypoxic pups (Table 1). It has previously been shown that the cerebellum is disproportionately affected by glucocorticoid treatment compared to brain and body size [30]. Similarly, the cerebellum is disproportionately affected under present conditions (Table 1). However, at P21 and P40, hypoxia-exposed mice showed ongoing cerebellar hypoplasia, despite catch-up in body weight and cerebral size (Table 1), with non-hypoxia-exposed littermates (Fig. 1b, c; P40 Nx vs. Hx, *p* = 0.018; IGL outlined for clarity).

**Table 1:** Body weights of P4 pups or P21 mice reared in normoxia or hypoxia

Age	*n*	Treatment	Body weight (g)	Brain weight (g)	Forebrain weight (g)
P4	3	Nx + veh.	2.66 ± 0.241	0.20 ± 0.002	0.145 ± 0.0003
P4	3	Hx + veh.	2.15 ± 0.129	0.18 ± 0.001	0.1323 ± 0.011
P4	4	Hx + Pred	2.10 ± 0.115	0.17 ± 0.001	0.119 ± 0.016
P4	4	Hx + Pred + SAG	2.04 ± 0.114	0.18 ± 0.002	0.127 ± 0.006
P21	3	Nx + veh.	10.5 ± 2.04	0.28 ± 0.028	0.2012 ± 0.01042
P21	4	Hx + veh.	9.77 ± 2.07	0.26 ± 0.005	0.1967 ± 0.01155
P21	5	Hx + Pred	9.12 ± 1.80	0.29 ± 0.03	0.2030 ± 0.02215
P21	4	Hx + Pred + SAG	9.48 ± 2.26	0.27 ± 0.03	0.1920 ± 0.02828

Measurements of weights are all in grams, average ± standard deviation

*Nx* normoxia, *veh* vehicle, *Hx* hypoxia, *Pred* prednisolone

**Fig. 2:**
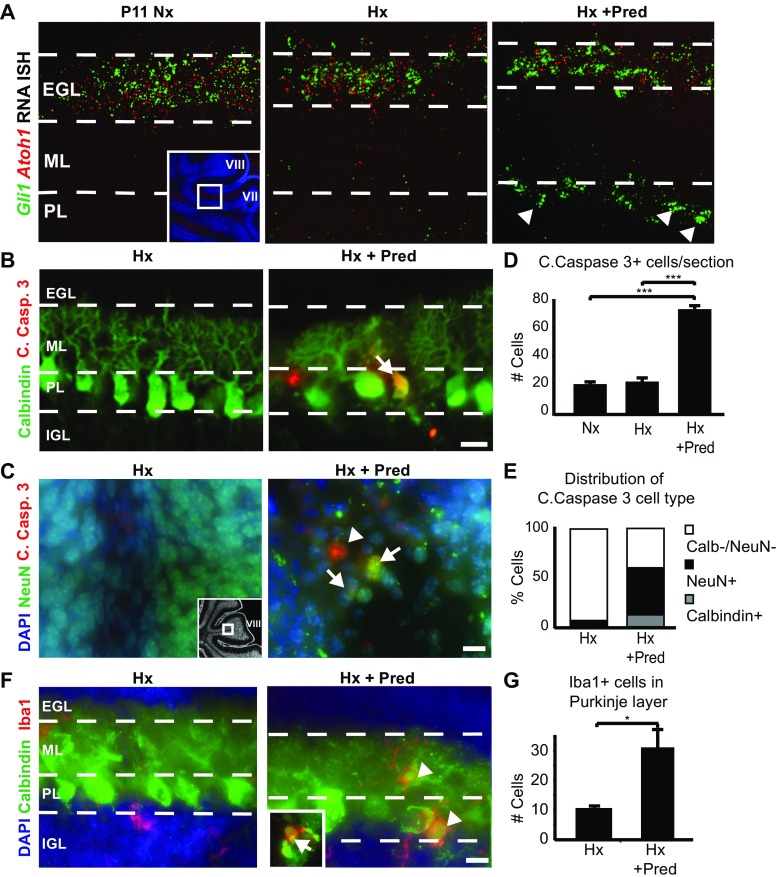
Glucocorticoid administration exacerbates chronic hypoxic injury to increase cerebellar damage through neuronal apoptosis. **a** Fluorescent in situ hybridization shows Shh signaling is decreased in animals reared in hypoxia (P11 Hx), and is exacerbated under dual injury hypoxia plus prednisolone (P11 Hx + Pred). Insert, diagram showing area of high magnification. *EGL* external granule layer, *ML* molecular layer, *PL* Purkinje layer. Arrow denotes Gli1 signal in the PL. **b** Representative images of the Purkinje cell layer (PL) showing absence or presence of Purkinje cells (Calbindin, red) undergoing apoptosis (C. Casp. 3, green) in hypoxic (P11 Hx) or hypoxic plus prednisolone (P11 Hx + Pred) samples at the end of the chronic hypoxic rearing. Arrow denotes double-labeled cell. *EGL* external granule layer, *ML* molecular layer, *PL* Purkinje layer, *IGL* internal granule layer. Scale bar, 10 μm. **c** Representative images of the IGL within lobule VIII of the cerebellum showing absence or presence of cerebellar granule neurons (NeuN, green) undergoing apoptosis (C. Casp.3, red) in Hx or Hx + Pred conditions at P11. Arrows denote double-labeled cells, arrowhead denotes C. Casp.3 only cells. *DAPI* blue for nuclear counterstain. Scale bar, 10 μm. Insert, low magnification image of cerebellum showing region of interest in lobule VIII from P11 sagittal cerebellar vermis. **d** Quantification of C. Casp.3 positive cells in normoxic (Nx), hypoxic (Hx), or hypoxic plus prednisolone (Hx + Pred) conditions. Nx, average counts = 20.75 ± 1.65 cells; Hx, average counts = 22.6 ± 2.54 cells; Hx + Pred, average counts = 53.86 ± 1.81 cells. **e** Distribution of apoptotic cells in Hx or Hx + Pred samples. Hx, Calb−/NeuN− = 93%, NeuN+ = 5%, Calbindin+ = 1%. Hx + Pred, Calb−/NeuN− = 40%, NeuN+ = 46.8%, Calbindin+ = 12.2%. **f** Representative images of Iba1-positive microglia in the Purkinje layer form lobule VII. Dashed lines denote layer borders similar to (**a**). Arrowheads denote Iba1+ cells in PL. Insert, example of a double-positive cell for Calbindin and Iba1. Scale bar, 10 μm. **g** Quantification of Iba1-positive cells in the PL in Hx and Hx + Pred samples. Hx, average counts = 9.5 ± 0.5 cells; Hx + Pred, average counts = 30.3 ± 7.5 cells. For quantification, mean + SEM; *n* ≥ 3 experiments/condition; *p < 0.05, ***p < 0.001, ANOVA with Tukey’s post-hoc correction

Cerebellar hypoplasia could possibly be due to cell death or decreased proliferation in the external granule layer (EGL). We first analyzed cleaved caspase 3 (Casp3) as a marker of apoptosis and phospho-histone H3 (pH 3) as a marker of mitosis. While there was no significant difference for Casp3 counts between hypoxic and normoxic mice (Fig. 2c), pH 3 counts were significantly decreased in CGNPs of at P11 (Fig. 1d, e; Nx vs. Hx, *p* = 0.002). As CGNP proliferation is driven by Shh signaling, we investigated whether decreased proliferation was a result of Shh downregulation. As shown (Fig. 1f, g), we found that levels of the Shh targets Patched 1 (Ptch1), N-myc, and cyclin D1 (CCD1) were downregulated in whole cerebella lysates from P11 hypoxic pups. To confirm downregulation of Shh signaling specifically in CGNPs, we collected protein lysates from primary cultures incubated in a 1% O_2_ hypoxic incubator for 24 h, or cultures treated with DMOG to upregulate HIF signaling (Supplementary Fig. 1). We found decreased levels of Ptch1 and increased levels of Gli3, which act as a repressor of Shh signaling, under DMOG or hypoxic conditions compared to control conditions. Treating our cultures with ShhN, which mimic the proliferating EGL in vivo, increased target protein levels of Gli1, N-myc, and Ptch1 as expected.

We have previously shown that Sonic hedgehog-Smoothened agonist (SAG) administration in postnatal pups crosses the blood–brain barrier and upregulates a Shh signaling (Gli-luciferase) reporter in the cerebellum [29]. As shown (Fig. 1e–g), SAG given daily from P3–11 resulted in a significant increase in cells in mitosis in the EGL only under hypoxic conditions (Fig. 1e; Hx vs. Hx + SAG, *p* = 0.002; Nx vs. Nx + SAG, *n.s.*) and Shh target protein levels from whole cerebellar lysates (Fig. 1f, g). Although chronic hypoxia may affect many cerebellar populations to contribute to hypoplasia, these findings suggest that hypoxia causes cerebellar hypoplasia in part through downregulated Shh pathway activity in CGNPs that can lead to decreased proliferation.

### Glucocorticoid Administration Plus Chronic Hypoxia Exacerbates Cerebellar Injury

Human preterm infants with chronic lung disease may become exposed to intermittent hypoxemia and postnatal glucocorticoids [7, 29]; thus, we investigated the combined impact of hypoxic rearing and GC administration on cerebellar development (Fig. 1a). We used prednisolone based on our previous finding that it causes cerebellar injury and can be rescued by SAG administration, which promotes upregulation of the protective enzyme 11βHSD2 in CGNP [29]. We first investigated Shh signaling under injury conditions specifically in the EGL and CGNPs. Fluorescent in situ hybridization shows that the Shh downstream target *Gli1* is reduced in P11 cerebella in CGNPs, which express *Atoh1* (Fig. 2a, left and center images) in both chronic hypoxia (Hx) and chronic hypoxia combined with daily prednisolone (Hx + Pred) administration from P3 to 11. This could be due to gene downregulation or reduced population of CGNPs. Unexpectedly, we observed ectopic expression of *Gli1* level in the Purkinje cell layer (PL, Fig. 2a, right; Supplementary Fig. 4) only in the condition of dual injury (Hx + Pred), which might be attributable to *Gli1* expression in either Bergmann glia or invading microglia (described below and in discussion).

As shown (Fig. 2b, d), we found increased apoptosis via Cleaved Caspase 3 (Casp3) staining in the P11 cerebellum of animals subjected to hypoxia plus prednisolone (Nx vs. Hx, n.s.; Nx vs. Hx + Pred, *p* < 0.001). Apoptotic cells included Calbindin + Purkinje cells and NeuN + cerebellar granule neurons (CGNs) of the internal granule layer that together comprised over half of the total Casp3+ cells (Fig. 2b–e). Additionally, we observed abnormalities in the Purkinje cell layer, including arborization defects and cell soma enlargement (Fig. 2b, f, Supplementary Figs. 2 and 3). In posterior lobules 7–9, we observed areas of markedly depleted Calbindin signal indicative of massive Purkinje cell loss (Supplementary Fig. 2). Inflammation was greater in the dual injury model, which showed an increase in activated Iba1+ microglia that invaded into the Purkinje layer, with colocalization of Iba1 and Calbindin noted in a few Purkinje cells (Fig. 2f and insert, g; Hx vs. Hx + Pred, *p* < 0.05). It is not clear whether increased Iba1+ microglia result from the dual insults themselves or increased cell death and activation to clear debris. In contrast, animals exposed to hypoxia alone showed fewer than 5% of neuronal cells that were Casp3+ (Fig. 2e). Together, these data indicate that glucocorticoid administration plus hypoxia exacerbates cerebellar injury targeting both CGNs and Purkinje cells.

### SAG Is Neuroprotective Against Cerebellar Hypoplasia and Purkinje Cell Loss After Complex Injury

**Fig. 3:**
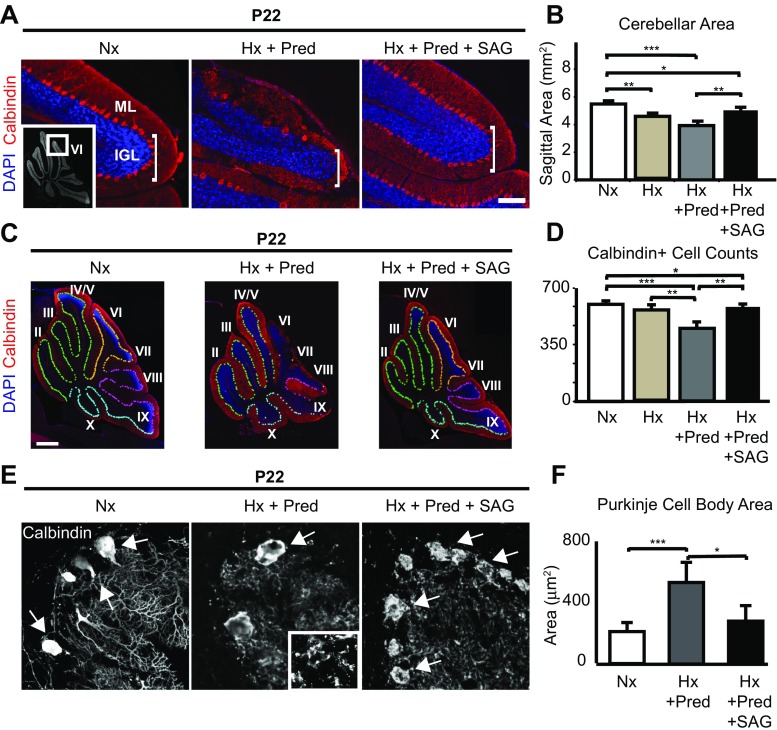
Chronic hypoxia plus prednisolone result in increased cerebellar hypoplasia and Purkinje cell loss, and SAG can partially rescue both phenotypes. **a** Representative images of lobule 6 in the cerebellar vermis showing the molecular layer (ML) and internal granule layer (IGL) from normoxic (Nx), hypoxic plus prednisolone (Hx + Pred), or hypoxic plus prednisolone and SAG (Hx + Pred + SAG) brain samples. Purkinje cells stained in red (Calbindin), and nuclei counterstained in blue (DAPI). Note size difference of IGL among the samples. Insert, location of lobule 6 as shown in whole cerebellum at lower magnification. Scale bar, 50 μm. **b** Quantification of cerebellar vermis area in Nx, Hx + Pred, and Hx + Pred + SAG conditions. Nx = 5.49 ± 0.104 mm^2^ (*n =* 3), Hx = 4.56 ± 0.120 mm2 (*n =* 3), Hx + Pred = 3.93 ± 0.324 mm^2^ (*n =* 5), Hx + Pred + SAG = 5.03 ± 0.062 mm^2^ (*n =* 5). **c** Representative images of whole cerebella showing extent of Purkinje cell damage in Nx, Hx + Pred, and Hx + Pred + SAG brains at P22. Purkinje cells stained with Calbindin (red), nuclei counterstained with DAPI (blue). Pseudocolors show demarcation of Purkinje cells from anterior (light green) through medial (orange/pink) and posterior (light blue) regions. Scale bar, 1 mm. **d** Quantification of Calbindin positive cells in Nx, Hx, Hx + Pred, or Hx + Pred + SAG. Nx = 604 ± 13 cells (*n =* 3), Hx = 562 ± 10 cells (*n =* 5), Hx + Pred = 452 ± 27 cells (*n =* 6), Hx + Pred + SAG = 535 ± 7 cells (*n =* 5). For quantification, mean + SEM. **e** Representative images of high-power Calbindin-positive Purkinje cells in Nx, Hx + Pred, or Hx + Pred + SAG brains at P22 showing extent of cell and dendritic damage. Arrows indicate Purkinje cell soma. Insert, higher power magnification of molecular layer showing disruption of dendrites and absence of Caspase 3. **f** Quantification of Purkinje cell body area. Nx = 216 ± 27.4 μm^2^, Hx + Pred = 551 ± 60.9 μm^2^, Hx + Pred + SAG = 290 ± 46.9 μm^2^. For each condition, *n =* 3 brains, average of 25 cells. *p < 0.05, **p < 0.01, ***p < 0.001, ANOVA with Tukey’s post-hoc correction

SAG is neuroprotective for the neonatal cerebellum in mouse models of glucocorticoid injury [29] and Down syndrome [56]. We next investigated whether SAG was beneficial in the setting of complex injury using two administration paradigms (Fig. 1a). In the first procedure, SAG (20 mg/kg body weight) was administered daily with prednisolone from P3 to P11. In the second case, we gave only a one-time dose of SAG (20 mg/kg body weight) after injury at P11. In both cases, we analyzed the cerebellum at P22. As shown (Fig. 3a, b), pups reared under hypoxia + prednisolone conditions showed greater vermian cerebellar hypoplasia and tissue damage, notably a reduction in the number of lobules, than littermates subjected to hypoxia only (*p* < 0.001). However, hypoplasia was significantly improved with SAG treatment (*p* < 0.01). As shown (Fig. 3a, b), SAG given as either in a chronic regimen or an acute one-time administration was effective at promoting partial rescue of cerebellar volume.

In addition, we found that SAG protected against Purkinje cell death. Purkinje cell survival after hypoxia + prednisolone was significantly (*p* < 0.001) compromised at P22 compared to animals that received hypoxia alone (Fig. 3c, d), which was especially prominent in posterior cerebellar lobules 6–10 (Fig. 3a, c). In addition to a decrease in Purkinje cell number, hypoxia + prednisolone caused a disruption in the Purkinje layer and dendritic arborization that is visible at P11 (Figs. 2f and 3e, middle panel insert) and began between P7 and P9 (Supplementary Fig. 3). By P22, cleaved caspase 3-positive cells were no longer detectable in the Purkinje layer. As shown (Fig. 3c, d), one-time SAG administration was able to partially rescue Purkinje cell loss (*p* < 0.01) and many of the surviving Purkinje cells showed a more normalized and less hypertrophic morphology (Fig. 3e, f). In contrast, SAG administration did not appear to rescue dendrite morphology (Fig. 3e).

These results demonstrate that hypoxia + prednisolone administration results in complex cerebellar injury, and that even a one-time dose of SAG at P11 post-injury partially protects the cerebellum from adverse impact. It seems likely normalization of CGNP proliferation with SAG is via the canonical Shh pathway [29, 30] (Fig. 1f, g), whereas the mechanisms resulting in Purkinje cell survival are less clear and require further study. It is possible that abnormalities are in part secondary to the decrease in CGNP cells as previously reported [19], while the loss of Shh activity resulting from hypoxia and prednisolone may influence late Purkinje cell maturation.

### Cellular Effects of HIF Pathway Plus Glucocorticoids in CGNPs

**Fig. 4:**
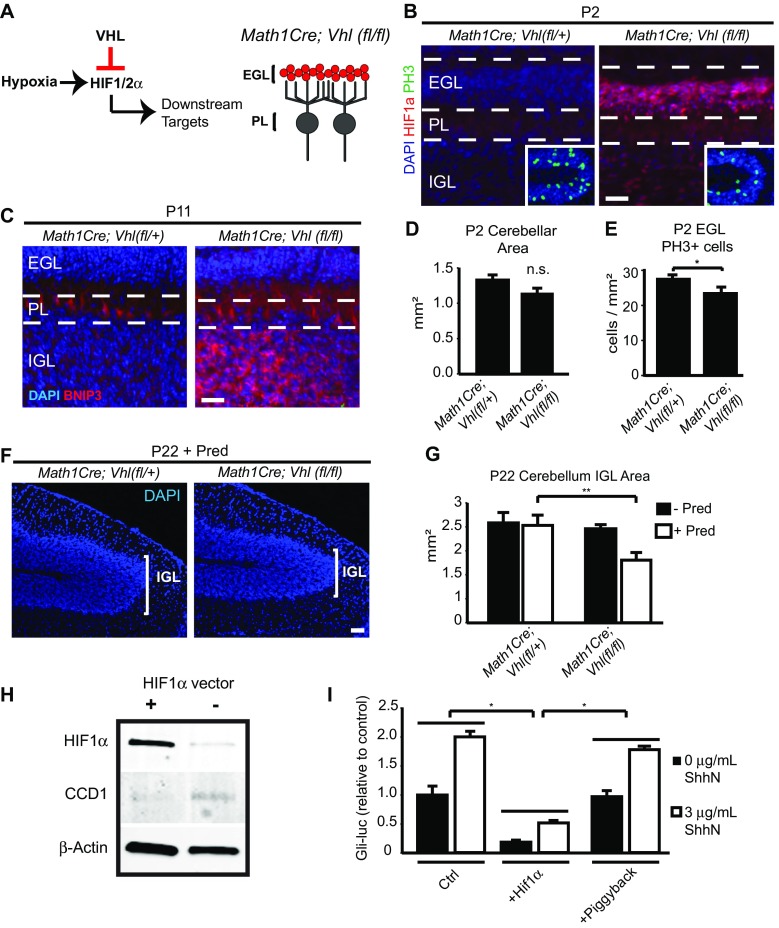
The HIF pathway plays a role in maintaining CGNP proliferation, and overactivation in CGNPs result in prednisolone-mediated cerebellar hypoplasia. **a** Right, schematic diagram showing HIF pathway. Left, schematic of cerebellar circuit highlighting CGNP-specific Cre recombination (red). *EGL* external granule layer, *PL* Purkinje cell layer. **b** Representative images of CGNPs in the external granule layer (EGL) and CGNs in the internal granule layer (IGL) with absence or presence of HIF1α (red). Insert, mitotic cells positive for PH3 (green) in the EGL. Nuclei counterstained with DAPI (blue). Scale bar, 50 μm. **c** P11 animals show increased expression of the HIF target BNIP3 in homozygous floxed animals only. Representative lobule 8 of cerebellar vermis. *EGL* external granule layer, *PL* Purkinje cell layer, *IGL* internal granule layer. Scale bar, 50 μm. **d** Quantification of cerebellar size at P2. *n.s.*, no significant difference. **e** Quantification of PH3+ cells in EGL at P2. *Math1Cre*;*Vhl*(*fl*/+) = 27.17 ± 0.437 cells/mm^2^, *Math1Cre*;*Vhl*(*fl*/*fl*) = 23.16 ± 1.40 cells/mm^2^. For quantification, *n* ≥ 3 per experiments, *p < 0.05, Student’s *t* test. **f** Representative images of lobule 6 in P22 brains receiving Pred administration from P3 to P11. Nuclei are counterstained with DAPI (blue) to visualize IGL. Scale bar, 50 μm. **g** Quantification of IGL cross-sectional area in P22 transgenic mice. *Math1Cre*;*Vhl*(*fl*/+) = 2.49 ± 0.264 mm^2^ (*n =* 3), *Math1Cre*;*Vhl*(*fl*/*fl*) = 2.34 ± 0.103 mm^2^ (*n =* 4), *Math1Cre*;*Vhl*(*fl*/+) + Pred = 2.44 ± 0.257 mm^2^ (*n =* 7), *Math1Cre*;*Vhl*(*fl*/*fl*) + Pred = 1.57 ± 0.190 mm^2^ (*n =* 6). **p < 0.01, ANOVA with Tukey’s post-hoc correction. For quantification, *n* ≥ 3 experiments per condition. **h** Transfection of HIF1α overexpressing vector in primary CGNP cultures, and representative Western for HIF1α and cyclin D1 (CCD1), with β-Actin used for normalization. **i** Primary CGNP cultures from the *Gli*-*Luciferase* reporter mouse line were transfected with HIF1α construct and assayed for luciferase activity 24 h later. Values depicted as relative to signal intensity in control condition. Ctrl = 1 ± 0.15 arbitrary units (au), Ctrl + Shh*n =* 1.99 ± 0.098 au, HIF1a = 0.179 ± 0.0311 au, HIF1a + Shh*n =* 0.51 ± 0.018 au, Piggyback = 0.961 ± 0.092 au, Piggyback + Shh*n =* 1.77 ± 0.054 au. *n =* 3 per condition. *p > 0.05, Student’s *t* test

We next investigated cell type-specific effects of HIF function plus Pred in conditional mutant transgenic mice. HIF1α is targeted for proteosomal degradation by the von Hippel Lindau (VHL) factor under normoxic conditions (Fig. 4a) [42, 43]. We first targeted CGNPs for HIF activation using *Math1Cre*;*Vhl*(*f*/*f*) transgenic mice (Fig. 4b; EGL layer in red). As expected, in normoxic mice, CGNPs showed strong HIF1α expression at P2. Furthermore, BNIP3, a target of HIF activation, was upregulated specifically in the IGL at P2 and P11 (Fig. 4c, data not shown), demonstrating HIF transcriptional activity. However, because the phenotype does not capture the full impact of hypoxia in CGNP, we conclude that other hypoxia-induced factors besides HIF or HIF signaling in other cells accounts for the full impact of hypoxia. HIF overactivation did not cause cerebellar hypoplasia, although there was a trend toward decreased cerebellar size at P2 that did not reach significance (Fig. 4d). HIF activation resulted in decreased CGNP proliferation at P2 (Fig. 4b insert, e; *p* < 0.05), which eventually normalized at later ages (data not shown). Basal BNIP3 expression in the Purkinje layer was also observed in both wild-type and transgenic conditions.

Although high HIF expression alone did not cause cerebellar hypoplasia, we hypothesized that HIF primed CGNPs for glucocorticoid-mediated injury, as previous reports link HIF activation with upregulation of the glucocorticoid receptor (GR) [57], and there can be crosstalk between hypoxia-dependent signals and glucocorticoid-mediated gene regulation [58]. Indeed, administration of prednisolone from P3 to 11 in *Math1Cre*;*Vhl*(*fl*/*fl*) animals caused significant (*p* < 0.01) decrease in the cross-sectional area in the vermis (Fig. 4f, g). These findings indicate that prednisolone administration combined with CGNP-specific HIF activation results in cerebellar hypoplasia.

VHL has other functions besides HIF1α degradation [59]. To further investigate HIF1α activity per se, we cultured CGNPs and transfected them with an overexpressing construct. Protein analysis revealed that HIF1α expression was greater in transfected conditions, and that the G1 cell cycle protein Cyclin D1 was downregulated (Fig. 4h). By using *Gli*-*luciferase* reporter mice [49], we found that transfected cultures showed lesser Shh activity in CGNPs (Fig. 4i). We conclude that HIF1α activity inhibits CGNP Shh activity and proliferation, and primes CGNPs for enhanced toxicity when exposed to glucocorticoids.

### Cellular Effects of HIF Pathway Plus Glucocorticoids in Purkinje Cell Neurons

**Fig. 5:**
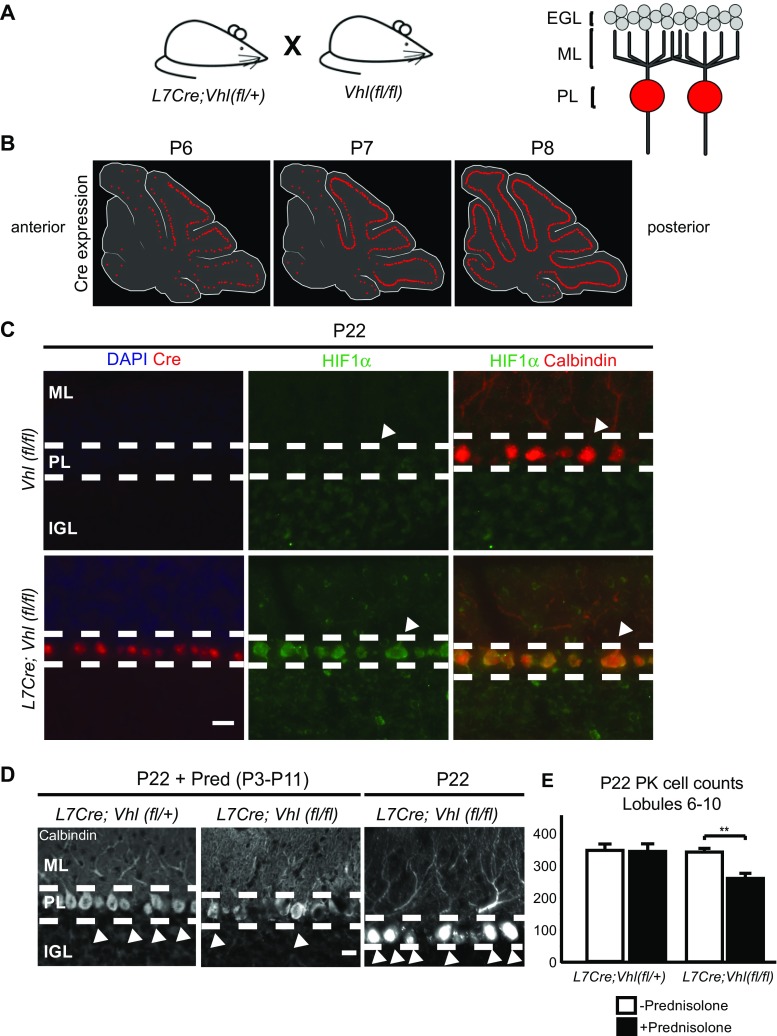
The HIF pathway plays a role in PK cell injury from prednisolone administration in *L7Cre*;*Vhl*(*fl*/*fl*) mice. **a** Right, schematic diagram showing transgenic mouse breeding to target Purkinje cells in *L7Cre*;*Vhl*(*fl*/*fl*) animals. Left, schematic of cerebellar circuit highlighting Purkinje-specific Cre recombination (red). *EGL* external granule layer, *ML* molecular layer, *PL* Purkinje cell layer. **b** Diagram highlighting timeline of *Cre* expression in P6 to P8 mouse pups. Note *Cre* expression turns on in posterior regions, specifically in lobules 6–9 of the cerebellar vermis, before anterior regions. **c** Representative images showing Cre (red, left column) and HIF1α (green) expression in the PL at P22 only in *L7Cre*;*Vhl*(*fl*/*fl*) animals. Note HIF1α colocalization with Calbindin (red, right column, denoted by arrowheads) expression in the PL. *ML* molecular layer, *PL* Purkinje cell layer, *IGL* internal granule layer. Scale bar, 20 μm. **d** Representative images showing loss of Calbindin + cells (arrowheads) in P22 *L7Cre*;*Vhl*(*fl*/*fl*) animals given daily Pred injections from P3 to P11. Dashed lines denote layer borders similar to (**c**). Scale bar, 10 μm. **e** Quantification of Calbindin + PK cells in posterior lobules. *L7Cre*;*Vhl*(*fl*/+) = 343.3 ± 19.6 cells (*n =* 3), *L7Cre*;*Vhl*(*fl*/*fl*) = 338.5 ± 23.5 cells (*n =* 3), *L7Cre*;*Vhl*(*fl*/+) + Pred = 339.2 ± 9.43 cells (*n =* 3), *L7Cre*;*Vhl*(*fl*/*fl*) + Pred = 255.8 ± 16.1 cells (*n =* 4). **p < 0.01, Student’s *t* test. For quantification, *n* ≥ 3 experiments per condition

The Purkinje cell is generally thought to be highly vulnerable to hypoxic injury in humans [3] and rodents [60]. Thus, we predicted that HIF1α stabilization in *Purkinje cell*-*specific Protein 2* (*Pcp2*/*L7*)-*cre*;*Vhl*(*fl*/*fl*) animals would have devastating effects (Fig. 5a). In these animals, L7 cre activity commences before birth and gradually becomes expressed in all Purkinje cells. As shown (Fig. 5b), cre expression is first detected in caudal cerebellum and becomes generally expressed in Purkinje cells by P8. As expected, HIF1α expression was specific to Purkinje cells in *L7cre*;*Vhl*(*fl*/*fl*) animals at P22 (Fig. 5c).

**Fig. 6:**
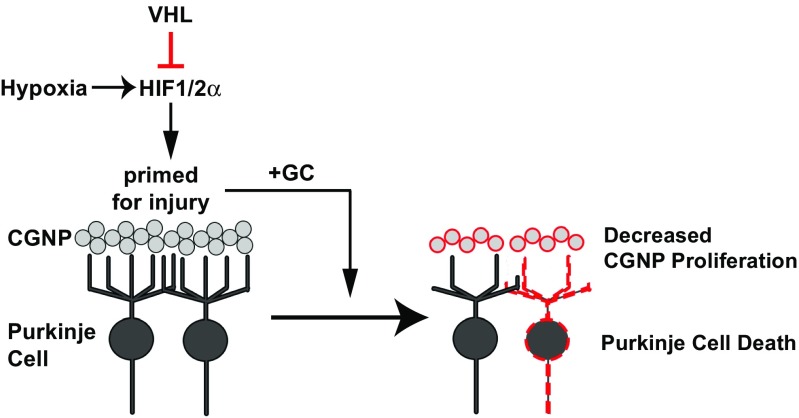
HIF activation promotes GC-mediated injury. Model of HIF activation in CGNPs or Purkinje cells, with as-yet unknown mechanisms leading to greater injury from glucocorticoid administration, and protection via SAG

Surprisingly, we saw no evidence of Purkinje cell loss in *L7cre*;*Vhl*(*fl*/*fl*) animals despite strong upregulation of HIF1α. However, when compounded with prednisolone administration from P3 to 11, analysis at P22 revealed significant (*p* = 0.0079) Purkinje cell losses (Fig. 5d, e). We note that the caudal cerebellar lobules were the most affected, consistent with earlier cre driver activity in this region (Fig. 5b). Despite this reduction in Purkinje cell number, cerebellar size was unaffected (*L7Cre*;*Vhl*(*fl*/+) *=* 5.25 ± 0.037 mm^2^, *n =* 6; *L7Cre*;*Vhl*(*fl*/*fl*) = 5.20 ± 0.146 mm^2^, *n =* 4, n.s.; data not shown); further studies are needed to determine timing of death. Thus, HIF activation primes serious Purkinje cell injury when they are exposed to glucocorticoids. Together, these transgenic animal data indicate a role for HIF signaling to potentiate glucocorticoid-mediated injury in two neuronal populations of neonatal mouse cerebellum (Fig. 6).

## Discussion

About 13% of births in the USA occur preterm and due to advances in neonatal life support [1], larger numbers of these children grow up with complications of prematurity such as cerebral palsy and behavioral and cognitive problems. Common structural abnormalities in the brain of former preterm infants include white matter lesions and cerebellar hypoplasia. Clinically, postnatal glucocorticoids are used in the NICU to treat hypotension and chronic lung disease [7, 61, 62], but glucocorticoid treatment is associated with cerebellar injury and neurodevelopmental deficits, as well as disruption of the hypothalamo-pituitary-adrenal (HPA) axis [26, 63]. Chronic lung disease is an independent risk factor for neurocognitive problems and cerebellar hypoplasia. Our findings suggest interactions of glucocorticoid and hypoxia/HIF signaling pathways act in cell-type specific ways to adversely impact preterm cerebellar development. The combination of hypoxia and glucocorticoid exacerbated Purkinje cell death and cerebellar hypoplasia. Interestingly, such complex injury was assuaged by a single dose of SAG days after insult.

### Hypoxia Causes Shh Pathway Downregulation and Permanent Cerebellar Hypoplasia

Chronic lung disease, which can cause intermittent hypoxemia, is associated with cerebellar hypoplasia in human infants. We observed that chronic hypoxia caused cerebellar hypoplasia, which likely reflects inhibition of Shh pathway and decreased proliferation in CGNPs. Cerebellar size differences were evident as early as 24-h post-exposure to hypoxia in mice, with the difference becoming greater after 7 days. This cerebellar hypoplasia was evident at P40 demonstrating permanent damage. In contrast to CGNP, Purkinje cell numbers were unaffected by hypoxia alone.

The Shh pathway drives CGNP proliferation [12, 15–17, 19], and the Shh target genes N-myc, Ptch1, and CCD1 were downregulated when postnatal pups were subjected to hypoxia. This may be due to the HIF-pathway, since primary CGNP cultures overexpressing HIF resulted in decreased CCD1 and Gli1 transcription and decreased proliferation. In vivo, we observed a correlate at P2 with decreased CGNP proliferation in *Math1Cre*;*Vhl*(*fl*/*fl*) pups; however, this normalized at later stages and these animals did not have permanent cerebellar hypoplasia. These findings suggest that Shh is likely the target of hypoxia-induced cerebellar hypoplasia, and that a compensatory mechanism might operate in vivo to counteract pure HIF1α stabilization in normoxic pups after P2.

### HIF Signaling Primes CGNP and Purkinje Cells for Exacerbated Glucocorticoid-Induced Injury

Postnatal glucocorticoids such as dexamethasone, prednisolone, and corticosterone are used to treat hypotension and chronic lung disease in the NICU [7, 61, 62]. When we combined hypoxic rearing with prednisolone administration, we observed exaggerated cerebellar hypoplasia at P22 and significant Purkinje cell death compared to either condition alone. We used *Math1Cre*;*Vhl*(*fl*/*fl*) or *L7Cre*;*Vhl*(*fl*/*fl*) transgenic mice to specifically target *VHL* loss-of-function in CGNP or Purkinje cells, respectively, to better understand how HIF signaling and glucocorticoids might act in cell type-specific ways. While *Math1Cre*;*Vhl*(*fl*/*fl*) did not show long-term cerebellar abnormalities, the addition of prednisolone caused cerebellar hypoplasia with long-term consequences to the IGL. Prednisolone in *Math1Cre*;*Vhl*(*fl*/+) or wild-type animals did not result in this reduction; this is in contrast to previous work [30], possibly due to delayed administration of prednisolone, starting at P3 instead of P0. Thus the impact of increased HIF signaling in CGNPs may be priming the cerebellum for a second insult, in our case, prednisolone. We found expression of the HIF target BNIP3 in *Math1Cre*;*Vhl*(*fl*/*fl*) animals at P11 in the EGL but not IGL (Fig. 4c). Thus, proliferation-related effects of HIF on CGNPs most likely occur at earlier timepoints. This suggests that there are two different threshold points for prednisolone-induced injury in our transgenic models. We observe early HIF activation in the EGL before P11, and a continuous HIF signaling in the Purkinje cells that occurs past P11, as seen by BNIP3 in the Purkinje cells. In *L7Cre*;*Vhl*(*fl*/*fl*) animals, prednisolone administration caused cell death of the Purkinje cell population by P22. How the HIF-pathway contributes to glucocorticoid-mediated exaggerated injury remains unclear but may be related to activation of glucocorticoid receptor signaling by HIF [57, 64–66]. Our experiments showed a strong additive effect of hypoxia and prednisolone on degree of injury, dependent on the HIF pathway (Fig. 6).

### Neuroprotective Effects of Smoothened Agonist Against Complex Neonatal Cerebellar Injury

Based on previous studies showing that Smoothened agonist (SAG) can sustain cerebellar growth in the face of exogenous insults and in a Down syndrome mouse model [29, 56], we tested SAG in the setting of hypoxia-prednisolone combinatorial injury. SAG is effective in preventing glucocorticoid-induced cerebellar neurotoxicity in neonatal mice in an 11βHSD2-dependent manner [29, 30]. Prednisolone is an 11βHSD2 sensitive glucocorticoid and is the reason we chose to use this compound versus dexamethasone, which has been used in other studies of glucocorticoid-mediated injury [67–71]. Because SAG is a potent activator of the Shh pathway [52], there is theoretical risk of tumor formation (e.g., medulloblastoma) [72, 73]. However, previous studies are reassuring and show that SAG is non-tumorigenic [29]; animals studied here also showed no ill effects from SAG with normal gains in body weight.

When we administered SAG either chronically from P3 to 11 or as a one-time dose at P11, we saw increased CGNP proliferation and normalized cerebellar volume despite prednisolone + hypoxia exposure. The beneficial effects of SAG in CGNPs may be due to inactivation of prednisolone itself and potentially higher levels of Shh pathway activation, which would act to support CGNP proliferation. In a Down syndrome mouse model (Ts65Dn), one-time SAG administration rescued both cerebellar morphology and phenotypes associated with hippocampal deficits [56].

Surprisingly, SAG was also able to rescue Purkinje cell loss, although the protective mechanism is unclear. Purkinje cells appeared hypertrophic at P22 following hypoxia and GC administration. With SAG administration, Purkinje cell numbers were normalized and they did not show hypertrophy. We found arborization defects from Purkinje cells starting between P7 and P9, and reduced Purkinje cell number by P22. Zonouzi et al. [47] previously reported that chronic hypoxia resulted in reduced arborization by P7 without reducing Purkinje cell number. It is possible that the 2-day delay in aberrant arborization in our experiments is due to genetic strain differences or different markers: in the case of Zonouzi et al., they used transgenic NG2DsRed or GAD65-GFP mice of mixed genetic background. We observed ectopic *Gli1* upregulation in the Purkinje layer that was specific to the case of hypoxia plus prednisolone. Although further studies are needed to confirm which cell population is expressing *Gli1*, many *Gli* transcripts are observed outside the borders of Calbindin + Purkinje cells, suggesting the possibility that ectopic *Gli1* expression occurs in Bergmann glia or invading microglia under these conditions. In any case, SAG might rescue Purkinje cell death via trophic effects on dendrites. Alternatively, SAG might protect the cerebellum via anti-inflammatory roles and potentially promoting blood-brain barrier (BBB) integrity [74–76]. It is also possible that Bergmann glia are benefitted by SAG or that it might protect Purkinje dendrites in the molecular layer. Indeed, a recent study shows that Shh signaling is necessary to maintain normal Bergman glial phenotype [77].

## Conclusion

In summary, we found that the combination of hypoxia and GC administration, which modulates the Shh pathway, reduced CGNP proliferation and resulted in increased apoptosis of Purkinje cells. In prior work, we showed that GC signaling inhibited the Shh pathway in CGNPs [30]. In our hypoxic model, we have found that hypoxic conditions upregulate GR, suggesting a working model that hypoxia/HIF upregulates GR signaling and downregulates Shh targets. We found that even a one-time dose of SAG following injury was sufficient to confer substantive rescue of cerebellar damage. Is protecting Purkinje cells and cerebellar volume (a strong indicator of CGN number) sufficient to improve neurological function? While this seems an obvious conclusion, further studies are needed to establish this point. SAG also has been shown to positively affect hippocampal function in a mouse model of Down syndrome [56]. Our studies indicate that hypoxia/HIF + postnatal glucocorticoid administration act on distinct cellular pathways to cause cerebellar injury. They further suggest that SAG is neuroprotective in the setting of complex neonatal cerebellar injury, adding to the body of data that SAG could prove a useful therapeutic agent in selected clinical settings.

## Electronic supplementary material

Supplementary Figure 1 Disrupted Shh signaling in CGNPs under hypoxic incubation or DMOG. Representative immunoblots showing effect of HIF activation by DMOG or 24 h hypoxia in the absence or presence of Shh on the Shh target genes Gli1, Gli3, N-myc, and Patched1. (JPEG 64 kb)

Supplementary Figure 2 Purkinje cell loss under Hypoxia + Prednisolone. High-power image (63X) showing PK layer in lobule 7. Green, Calbindin-positive Purkinje cells, red, Iba1. Scale bar, 20 μm. (PDF 1026 kb)

Supplementary Figure 3 Arborization defects from Hypoxia + Prednisolone in Purkinje cells begin around P9. P7 and P9 cerebella stained for Calbindin show Purkinje cell arborization under hypoxia or hypoxia + prednisolone. EGL, external granular layer; PL, Purkinje cell layer; IGL, internal granular layer. Scale bar, 50 μm. (PDF 3489 kb)

Supplementary Figure 4 Ectopic expression of Gli1 in Purkinje layer. High magnification image showing Gli1 fluorescent ISH and Calbindin immunopositive cells in Purkinje layer from a P11 Hx + Pred cerebellum. Note the Gli1 expression is absent from Calbindin + Purkinje cells. (PDF 555 kb)

## Abbreviations

SAG: Smoothened agonist
Shh: Sonic hedgehog
HIF: Hypoxia-inducible factor
CGNP: Cerebellar granule neuron precursor
VHL: von Hippel Lindau factor
