# Supplementary figures and images for: Sonic Hedgehog Agonist Protects Against Complex Neonatal Cerebellar Injury
Source: Cerebellum. 2017 Nov 13;17(2):213–27. doi: 10.1007/s12311-017-0895-0 (PMC5849674; doi:10.1007/s12311-017-0895-0)

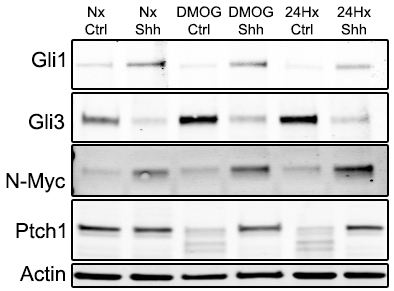

Supplement: Supplementary file 1 — Disrupted Shh signaling in CGNPs under hypoxic incubation or DMOG. Representative immunoblots showing effect of HIF activation by DMOG or 24 h hypoxia in the absence or presence of Shh on the Shh target genes Gli1, Gli3, N-myc, and Patched1. (JPEG 64 kb) [file 12311_2017_895_MOESM1_ESM.jpg]

# P11 Hx + Pred

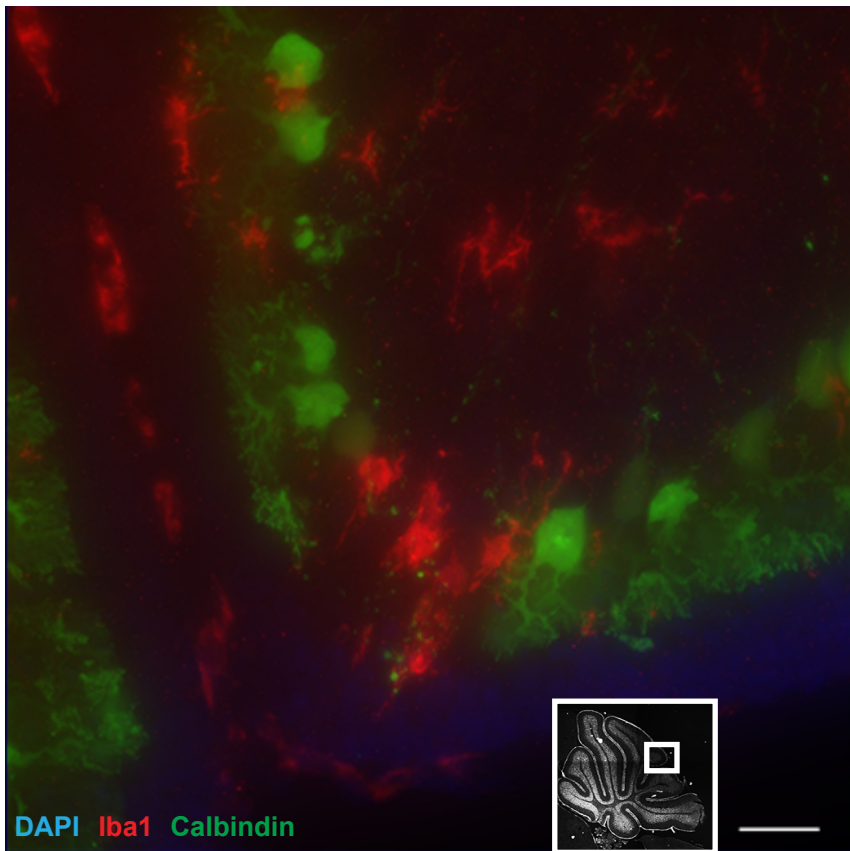

Supplement: Supplementary file 2 — Purkinje cell loss under Hypoxia + Prednisolone. High-power image (63X) showing PK layer in lobule 7. Green, Calbindin-positive Purkinje cells, red, Iba1. Scale bar, 20 μm. (PDF 1026 kb) [file 12311_2017_895_MOESM2_ESM.pdf]

P7

P9

-Prednisolone

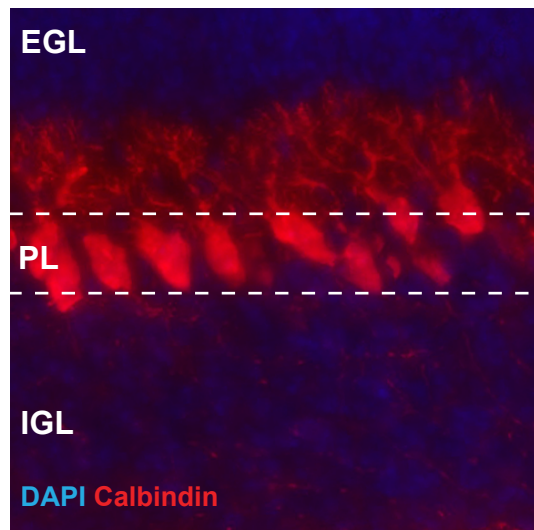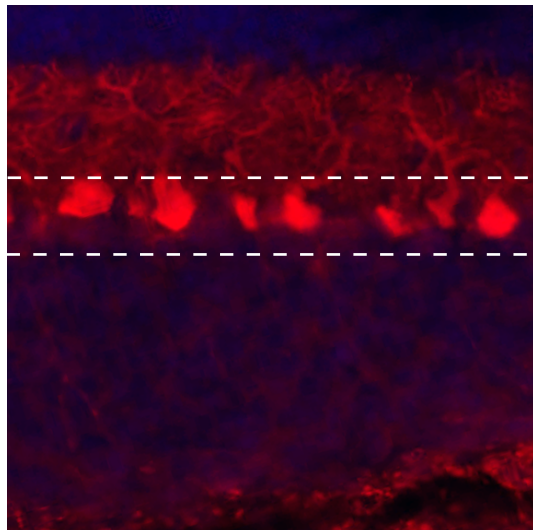

+Prednisolone

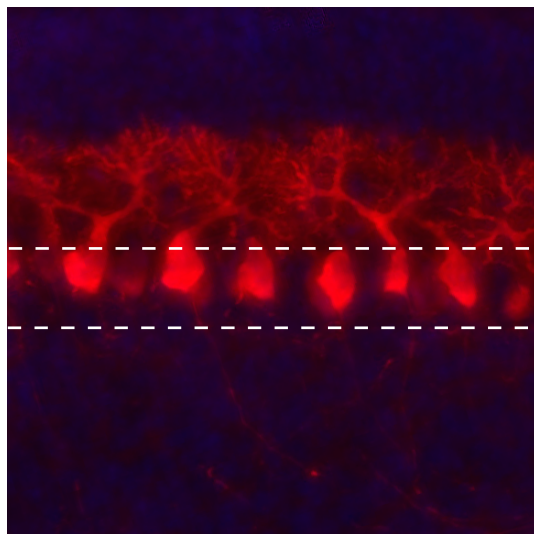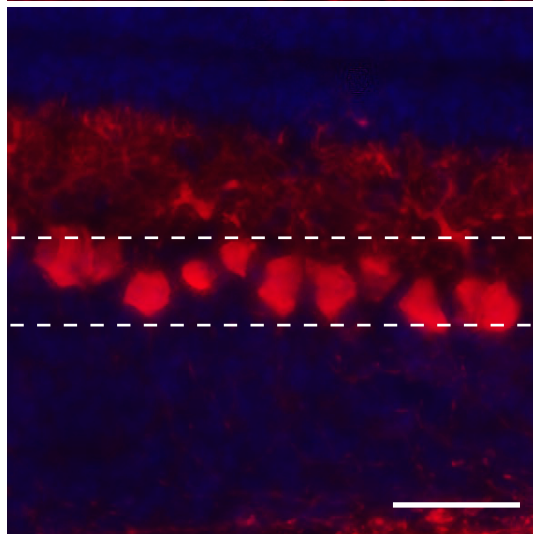

Supplement: Supplementary file 3 — Arborization defects from Hypoxia + Prednisolone in Purkinje cells begin around P9. P7 and P9 cerebella stained for Calbindin show Purkinje cell arborization under hypoxia or hypoxia + prednisolone. EGL, external granular layer; PL, Purkinje cell layer; IGL, internal granular layer. Scale bar, 50 μm. (PDF 3489 kb) [file 12311_2017_895_MOESM3_ESM.pdf]

# P11 Hx + Pred

DAPI *Gli1* Calbindin

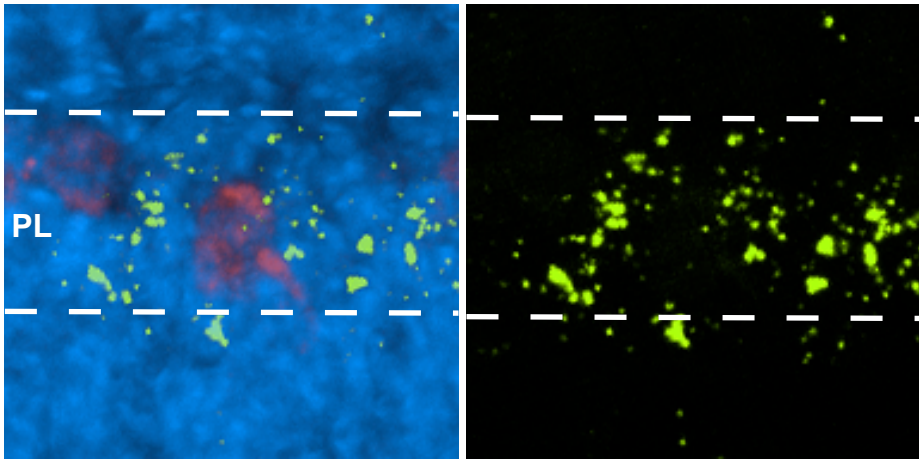

Supplement: Supplementary file 4 — Ectopic expression of Gli1 in Purkinje layer. High magnification image showing Gli1 fluorescent ISH and Calbindin immunopositive cells in Purkinje layer from a P11 Hx + Pred cerebellum. Note the Gli1 expression is absent from Calbindin + Purkinje cells. (PDF 555 kb) [file 12311_2017_895_MOESM4_ESM.pdf]
